# The characteristics of differentiated yeast subpopulations depend on their lifestyle and available nutrients

**DOI:** 10.1038/s41598-024-54300-9

**Published:** 2024-02-14

**Authors:** Michal Čáp, Zdena Palková

**Affiliations:** Department of Genetics and Microbiology, Faculty of Science, Charles University, BIOCEV, Prague, Czech Republic

**Keywords:** Microbial communities, Fungal biology

## Abstract

Yeast populations can undergo diversification during their growth and ageing, leading to the formation of different cell-types. Differentiation into two major subpopulations, differing in cell size and density and exhibiting distinct physiological and metabolic properties, was described in planktonic liquid cultures and in populations of colonies growing on semisolid surfaces. Here, we compare stress resistance, metabolism and expression of marker genes in seven differentiated cell subpopulations emerging during cultivation in liquid fermentative or respiratory media and during colony development on the same type of solid media. The results show that the more-dense cell subpopulations are more stress resistant than the less-dense subpopulations under all cultivation conditions tested. On the other hand, respiratory capacity, enzymatic activities and marker gene expression differed more between subpopulations. These characteristics are more influenced by the lifestyle of the population (colony vs. planktonic cultivation) and the medium composition. Only in the population growing in liquid respiratory medium, two subpopulations do not form as in the other conditions tested, but all cells exhibit a range of characteristics of the more-dense subpopulations. This suggests that signals for cell differentiation may be triggered by prior metabolic reprogramming or by an unknown signal from the structured environment in the colony.

## Introduction

Yeast populations grown in a well-shaken liquid medium have traditionally been considered homogeneous populations of uniform cells unless a specific program (e.g. sporulation) is triggered. In recent decades, however, it has become apparent that such a view is oversimplified, as evidenced by the isolation of distinct cell subpopulations from aged liquid cultures and from the complex environment of multicellular structures formed by yeast cells under static conditions in liquid media or on solid surfaces^1–7^. In the latter case, the differentiated cells are located in specific regions of the multicellular structures, such as cell layers in colonies or surface and “root” structures in biofilm colonies^1,4,5^.

Allen et al.^6^ isolated two distinct cell subpopulations with different density characteristics from several days old liquid shaken cultures of the yeast *Saccharomyces cerevisiae* grown in glucose-based complex medium (YPD). These two subpopulations differ in many respects in terms of their expression, metabolism and resistance to stresses. The more-dense cell fraction consists of larger cells (referred to as Q—quiescent cells) that are more resistant to heat shock, maintain their viability for a long period of time, produce less ROS, accumulate glycogen and trehalose, and have a higher respiration rate, compared to the second large subpopulation—the NQ (non-quiescent) cells. Population heterogeneity was also found during high-level ethanol production, where two cell subpopulations resembling Q and NQ cells were found^3^. Another cell differentiation has been described in aging yeast giant colonies (originating from a drop of cell suspension) and microcolonies, growing on non-fermentative complex agar (GMA), where two morphologically distinct subpopulations were identified^1,8^. These subpopulations form distinct and sharply separated layers, with the upper layers composed of U cells that are denser, larger, more stress resistant and more long-lived than the L cells in the lower layers. Thus, U cells resemble Q cells in some aspects, but detailed characterization of U and L cells showed that U cells, unlike Q cells, for example, have a lower respiratory capacity and increased glycolysis compared with L cells^9^. Two cell-types (termed inner and outer cells) were also found in microcolonies grown on solid YPD medium, showing some similarities with U/L cells based on transcriptomic data^5^. However, detailed physiological and biochemical characteristics of these cells are lacking.

Here, we asked how the properties of different stationary-phase cell types depend on the different living conditions of the yeast population (liquid medium versus structured colony environment) and on the different nutrient sources (fermentative glucose versus ethanol/glycerol respiratory medium). We compared quiescence-related traits and metabolic characteristics of differentiated cell subpopulations isolated from four different yeast cultures. In addition to U and L cells from colonies grown on respiratory agar medium GMA and Q and NQ cells from liquid glucose medium YPD, we separated and characterized differentiated cell subpopulations from YPD-grown colonies and from liquid cultures in GM medium.

## Results

### Seven cell subpopulations were isolated from four different populations

**Figure 1:**
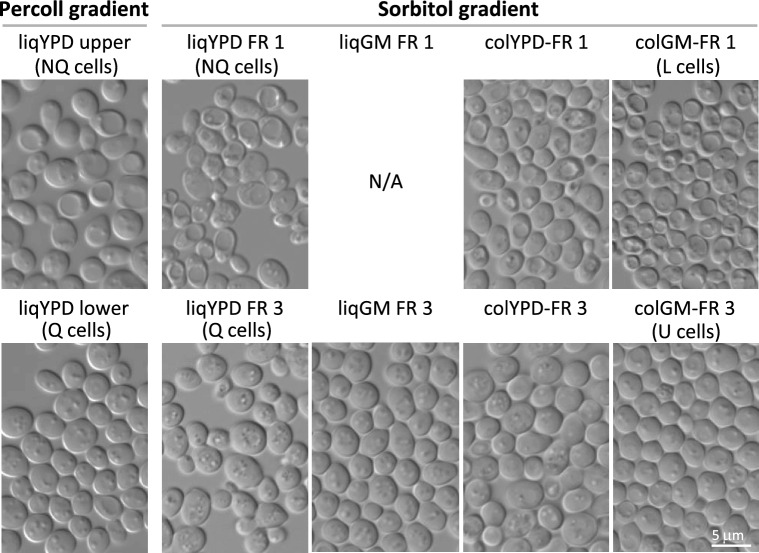
Cell subpopulations isolated from YPD- and GM-grown colonies (colYPD, colGM) and liquid cultures (liqYPD, liqGM). Subpopulations from liqYPD isolated by Percoll gradient centrifugation: the upper fraction contains less-dense cells (NQ cells) and the lower fraction contains more-dense cells (Q cells). Subpopulations isolated by sorbitol gradient centrifugation: Fraction 1 (FR 1) contains a less-dense subpopulation, and fraction 3 (FR 3) contains a more-dense subpopulation. The liqGM culture did not yield a less-dense subpopulation. liqYPD FR 1 and FR 3 (sorbitol gradient) are comparable to the upper and lower fractions of liqYPD (Percoll gradient).

Different methods have been used to isolate different cell types from liquid cultures and colonies. Allen et al.^6^ used isopycnic centrifugation in a Percoll gradient to isolate Q and NQ cells with different densities from liquid cultures in YPD, whereas we used rate-zonal centrifugation in a sucrose or sorbitol gradient to obtain U and L cells from colonies on GMA^1,9^. Both isolation methods yielded less-dense cell fractions (upper fraction in the Percoll gradient and fraction 1 in the sorbitol gradient) that contained morphologically similar cells with low buoyant density and clearly visible large vacuoles, and more-dense cell fractions (lower fraction in the Percoll gradient and fraction 3 in the sorbitol gradient) that contained robust cells with high buoyant density and no visible vacuoles (Fig. 1). To compare cells from different media and lifestyles (colonies and liquid cultures), we prefer rate-zonal centrifugation because it allows faster separation of cells (separation within 10 min compared with 1 h for isopycnic centrifugation), thus minimizing the time between sample collection and analysis. We first demonstrated that sorbitol gradient centrifugation allows efficient separation of subpopulations from liquid YPD cultures and that the two resulting subpopulations are identical to the subpopulations isolated by Percoll gradient centrifugation (i.e., Q and NQ cells) (Fig. 1). Sorbitol was used to avoid possible metabolic changes that could be induced by sucrose (e.g., glucose-mediated repression).

We then separated cells from 4 different yeast populations: (1) 7-day-old liquid culture in complex glucose medium (liqYPD), (2) 7-day-old liquid culture in glycerol–ethanol medium (liqGM), (3) 14-day-old colonies on complex glucose agar medium (colYPD), and (4) 14-day-old colonies on glycerol-ethanol agar medium (colGM). In 7-day-old liquid YPD cultures, cells are fully differentiated to Q and NQ^6^, whereas full U/L cell differentiation in giant colonies occurs later^1^, so we used 14-day-old colonies. Two visually distinguishable subpopulations of different densities were isolated from both colony populations (colGM and colYPD) and from liqYPD (Fig. 1). One of these subpopulations (fraction 1) has a lower density and visible vacuoles in DIC microscopy. The other subpopulation (fraction 3) consisted of larger cells with higher density and fewer visible vacuoles. In contrast, centrifugation of the liqGM population yielded only a more-dense cell type that morphologically resembled U cells from colGM. Even after prolonged cultivation (up to 40 days, data not shown), no further cell differentiation was observed in liqGM.

### Stress-related characteristics of isolated cell subpopulations

**Figure 2:**
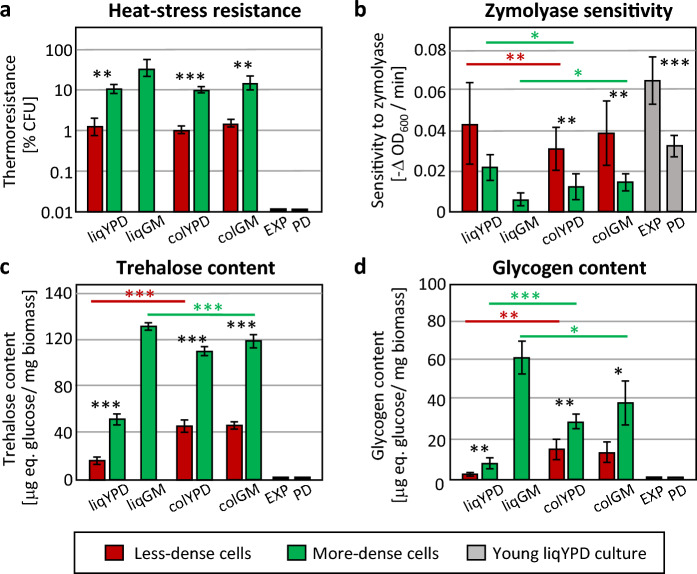
Stress sensitivity and storage carbohydrate accumulation as prominent quiescence markers in less-dense and more-dense cell subpopulations. EXP and PD are exponential and post-diauxic shift YPD grown cells, respectively. (**a**) Heat stress resistance, % CFU refers to the number of CFU before heat shock (100% CFU) in each sample. (**b**) Zymolyase sensitivity measured as decrease in OD_600_ in time during incubation in Zymolyase solution. (**c**,**d**) Content of storage carbohydrates trehalose (**c**) and glycogen (**d**), measured as the amount of glucose released after enzymatic digestion of biomass. Error bars indicate standard deviation from the mean, n = 3. Statistically significant differences between subpopulations from the same culture are indicated by asterisks. *indicates *p* value < 0.05, **p value < 0.01, ***p value < 0.001. Black asterisks refer to the comparison of subpopulations from the same cultivation, coloured asterisks refer to the comparison of corresponding subpopulations from different cultivations indicated by coloured horizontal lines (red colour, comparison of less-dense subpopulations; green colour, comparison of more-dense subpopulations).

Previous studies have shown that cells of the more-dense cell fraction (Q and U cells) have some characteristics attributed to stationary phase cells. They are resistant to various stress factors and accumulate the storage compounds glycogen and trehalose^1,6^. Therefore, we measured these characteristics in seven isolated cell types from the four growth conditions. For comparison, we used exponential cells from liquid YPD medium (culture before glucose depletion) and cells from postdiauxic liquid YPD culture (slow-growing culture after glucose depletion; ~ 16 h after inoculation). Heat shock resistance (30 min treatment at 52 °C) of the isolated cell types was assessed as survival in percent CFU (colony forming units) in a tenfold dilution drop test compared to the time before heat shock (0 min) (Fig. 2a). The heat shock resistance of less-dense cells (~ 1% survival) was approximately tenfold lower than that of more-dense cells (~ 10% survival) from colGM, colYPD, and liqYPD. More-dense cells from liqGM were more heat shock resistant than all other cell types. On the other hand, the heat shock resistance of exponential cells and postdiauxic cells (0.01% survival) was much lower than all other cell types, including less-dense cells. Similar results were obtained when cells were treated with the cell wall-degrading enzyme Zymolyase (Fig. 2b). The liqGM cells were more resistant than all other cell types. Less-dense cells were less resistant than more-dense cells in all cultivations, with resistance generally lower in liqYPD than in colYPD and colGM. Exponential cells were more sensitive than all less-dense cells, whereas postdiauxic cells were comparable to less-dense cells.

Glycogen and trehalose accumulation is another typical feature of cells in stationary phase^10^, and their content often correlates with cell stress resistance^11^. Similar to the previous characteristics, in all cases, more-dense cells had higher amounts of glycogen and trehalose than less-dense cells from the same population, and cells from liqGM contained the most storage carbohydrates (Fig. 2c,d). However, both cell types from liqYPD have much lower amounts of both saccharides (2–7×) than both colony populations and liquid culture in GM. A negligible amount of trehalose and glycogen was detected in exponential and postdiauxic cells from liquid YPD.

In summary, the more-dense cell subpopulations of all cultures were more resistant to stress and accumulated higher amounts of glycogen and trehalose than the less-dense subpopulations. In all cases, cells from liqGM were the most resistant and contained the greatest amounts of storage carbohydrates. In addition, all cells isolated from 7-day-old liquid cultures and 14-day-old colonies were more resistant and contained more storage carbohydrates than exponential cells and, with the exception of Zymolyase resistance, than post-diauxic cells.

### Metabolic characteristics of the cell subpopulations

**Figure 3:**
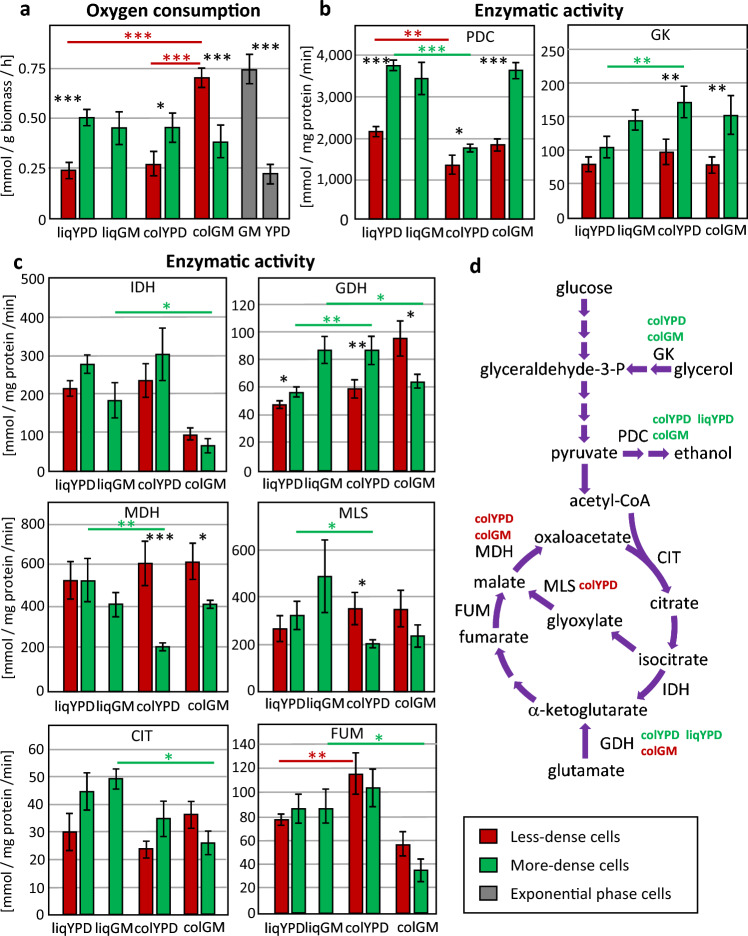
Respiration and enzymatic activities of less-dense and more-dense cell subpopulations. (**a**) Oxygen consumption rate of whole intact isolated cells as a measure of respiratory capacity in the presence of ethanol as substrate. (**b**) Enzymatic activities typical for U cells: pyruvate decarboxylase (PDC) and glycerol kinase (GK). (**c**) Enzymatic activities typical for L cells: citrate synthase (CIT), isocitrate dehydrogenase (IDH), glutamate dehydrogenase (GDH), fumarase (FUM), malate dehydrogenase (MDH), and malate synthase (MLS). (**d**) Scheme illustrating the analysed enzymatic activities in the context of central carbon metabolism. The cultivation conditions near enzymatic reactions indicate statistically significant differences between the subpopulations in the respective cultivation. Red and green colours indicate higher activity in the less- dense and more-dense subpopulation, respectively, compared to the other subpopulation of the same cultivation. Error bars indicate standard deviation from the mean, n = 3. Statistically significant differences between subpopulations from the same culture are indicated by asterisks. *indicates *p* value < 0.05, **p value < 0.01, ***p value < 0.001. Black asterisks refer to the comparison of subpopulations from the same cultivation, coloured asterisks refer to the comparison of corresponding subpopulations from different cultivations indicated by coloured horizontal lines (red colour, comparison of less-dense subpopulations; green colour, comparison of more-dense subpopulations).

Differences in metabolism have been described between Q/NQ cells and U/L cells^1,12^. For example, Q cell respiration is higher in liqYPD compared with NQ cells (i.e., higher respiration in the more-dense, stress-resistant subpopulation), whereas the respiratory capacity of colGM U cells is lower compared with L cells (i.e., lower respiratory capacity of the more-dense, stress-resistant subpopulation). We therefore compared the respiratory capacity of the isolated cell subpopulations, measured as oxygen consumption rate with ethanol as substrate (Fig. 3a). The more-dense cell subpopulations of liqYPD and colYPD showed higher oxygen consumption (2.1- and 1.6-fold, respectively) compared with the corresponding less-dense subpopulations. However, the less-dense L cells of colGM showed higher respiratory capacity than more-dense U cells, as shown previously^1^, and the highest respiratory capacity of all subpopulations analysed. The less-dense cells of liqYPD and colYPD had the lowest respiratory capacities.

Previous measurements of metabolic activities of enzymes involved in central carbon metabolism have shown that enzymes involved in the fermentation pathway are more active in more-dense U cells, whereas enzyme activities involved in the TCA cycle, glyoxylate cycle, and ketoglutarate-glutamate conversion are higher in less-dense L cells^9^. Therefore, we measured the activities of selected enzymes of these metabolic pathways (scheme Fig. 3d) in all more-dense and less-dense cell subpopulations.

The main differences in enzymatic activities between U and L cells include pyruvate decarboxylase (PDC, various isozymes, which convert pyruvate to acetaldehyde) and glycerol kinase (GK, Gut1p, which converts glycerol to glycerol 3-phosphate), which are higher in more-dense U cells (Fig. 3b). Accordingly, these two enzymatic activities were higher in more-dense cells than in less-dense cells under all conditions. The differences between more-dense and less-dense cells were less pronounced only for GK activity in liqYPD cells (*p* value > 0.05). liqGM cells showed similar activities of PDC and GK as the more-dense cells of colGM.

With regard to enzymatic activities, which were assumed to be higher in the less-dense L cells than in the more-dense U cells, two patterns can be distinguished (Fig. 3c). The first pattern was observed for the activity of glutamate dehydrogenase (GDH), which was higher in less-dense L cells than in more-dense U cells, but was lower in less-dense cells from both liqYPD and colYPD than in the corresponding more-dense cells (Fig. 3c). These differences are consistent with the differences in measured respiratory capacity (Fig. 3a). Similar trends in the profile of enzymatic activities between cell subpopulations were observed for citrate synthase (CIT) and isocitrate dehydrogenase (IDH), but were not statistically significant (*p* value > 0.05) (Fig. 3c). A different pattern was observed for malate dehydrogenase (MDH) and malate synthase (MLS) activities, which were higher in the less-dense cells of both colony populations, colYPD and colGM (MLS differences in colGM were less pronounced, *p* value > 0.05), but lower or indistinguishable in the less-dense cells in liqYPD compared to the corresponding more-dense cells (Fig. 3c). A similar, but statistically non-significant (*p* value > 0.05) trend was observed for fumarase (FUM) activity.

The activities of IDH and FUM were lower in colGM than in the other populations. The activities of IDH, GDH, FUM and CIT were higher in cells in liqGM than in the corresponding more-dense cells in colGM.

### Expression of marker genes

Previous studies have identified several marker genes that are predominantly expressed in a specific cell subpopulation. *ACS1*, *CIT1* and *NCE102*, encoding acetyl-CoA synthase, citrate synthase and a protein of unknown function, respectively, were selected as markers that are more highly expressed in more-dense Q cells than in less-dense NQ cells^12^. *ATO3*, *POX1*, and *MET17*, encoding ammonium transporter, peroxin, and an enzyme involved in methionine biosynthesis, respectively, are markers that are more highly expressed in more-dense U cells than in less-dense L cells, and *INO1* and *FBP1*, encoding enzymes involved in inositol phosphate biosynthesis and gluconeogenesis, respectively, are markers that are more highly expressed in less-dense L cells than in more-dense U cells (Ref.^1^ and our unpublished data). To compare the expression of these marker genes in all seven subpopulations, we measured the fluorescence intensity of GFP fused to the respective gene at its native locus, i.e. natively regulated in the respective population.

**Figure 4:**
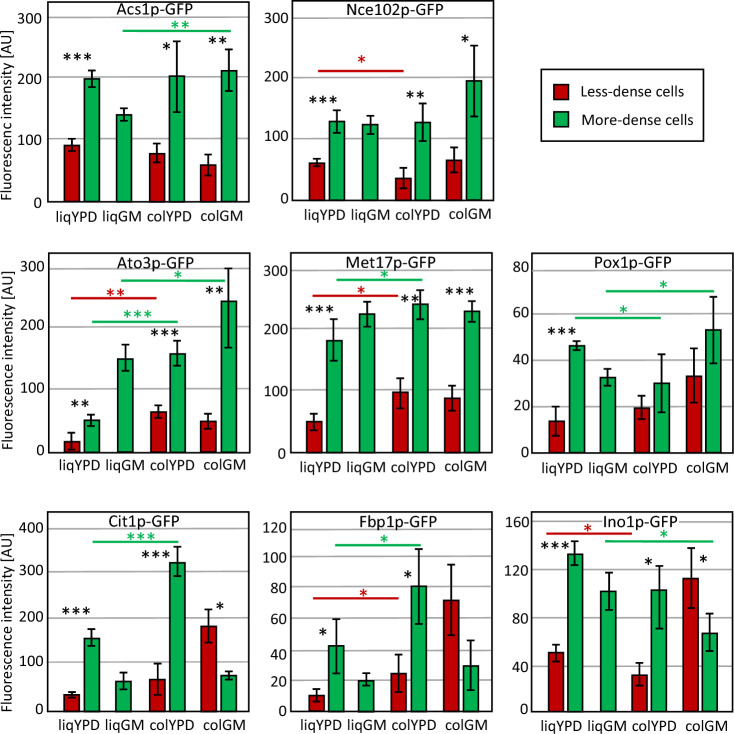
Fluorescence of selected GFP-labelled marker proteins in less-dense and more-dense cell subpopulations. Acs1p, Nce102p and Cit1p are markers of Q cells. Ato3p, Met17p and Pox1p are markers of U cells. Fbp1p and Ino1p are markers of L cells. Error bars indicate standard deviation from the mean, n = 3. Statistically significant differences between subpopulations from the same culture are indicated by asterisks. *indicates *p* value < 0.05, **p value < 0.01, ***p value < 0.001. Black asterisks refer to the comparison of subpopulations from the same cultivation, coloured asterisks refer to the comparison of corresponding subpopulations from different cultivations indicated by coloured horizontal lines (red colour, comparison of less-dense subpopulations; green colour, comparison of more-dense subpopulations).

Most markers for the more-dense cell subpopulations Q and U were more highly expressed in more-dense cells in all populations examined (Fig. 4). This was true for *ACS1* and *NCE102* (markers for more-dense Q cells) and *ATO3*, *MET17* and *POX1* (markers for more-dense U cells). The difference in Pox1p-GFP levels between U and L cells was less pronounced (*p* value > 0.05).

However, the more-dense Q-cell marker *CIT1* was only more highly expressed in the more-dense cells of the glucose-grown populations (liqYPD and colYPD) compared to the corresponding less-dense cells, whereas its expression was higher in the less-dense L cells of colGM compared to the more-dense U cells (Fig. 4). Similarly, the two L cell markers *INO1* and *FBP1* were, as expected, more highly expressed in less-dense L cells than in more-dense U cells (the difference for Fbp1p was less pronounced, *p* value > 0.05), but these genes were more highly expressed in the more-dense cells of the glucose-grown colonies and liquid cultures than in the corresponding less-dense cells (Fig. 4).

The expression of marker genes in liqGM cells was more similar to their expression in more-dense U cells of colGM than to their expression in less-dense L cells, with the exception of *POX1* and *INO1*, whose expression was more similar to their expression in less-dense L cells of colGM than to their expression in U cells. *FBP1* and *ATO3* were significantly more expressed in colony populations on both YPD and GM than in the corresponding cells in liqYPD (and liqGM in the case of *ATO3*).

## Discussion

In this study, we investigated the properties of 7 different stationary-phase cell subpopulations formed in ageing colonies and liquid cultures in two different media—fermentative YPD medium and respiratory GM medium. Upon entering stationary phase, cells acquire properties that help them maintain viability over an extended period of time under adverse conditions such as starvation or other stresses. These changes include strengthening of the cell wall, increased resistance to various stressors, increased accumulation of the storage and protective carbohydrates glycogen and trehalose, and increased amount of lipid droplets and decreased rates of transcription and translation compared to cells from exponentially growing cultures^10,13,14^. These features are typical of quiescent cells, which are defined as cells that exit the cell cycle into G_0_ phase and can re-enter the cell cycle under favourable circumstances^10,13^. In contrast, much less information is available on the differences between stationary-phase cells derived from populations developing under different conditions, although there are data clearly showing that different variants of quiescent cells can form^13,15–17^.

We show that under three of the four conditions tested, two large subpopulations of stationary-phase cells form (one more-dense and one less-dense), which can be distinguished visually and by characteristics commonly attributed to stationary-phase cells. These more-dense and less-dense types of cell subpopulations from three sources have common differences, but each cell type also has its specific features distinguishing it from others. Therefore each of the cell subpopulations has its own unique combination of characteristics, which is probably caused by a combination of influences such as nutrient sources and living conditions (colonies versus liquid conditions).

### Common differences between more-dense and less-dense cells are related to their stress resistance

In all three cultures (colonies on GM and YPD, and liquid culture in YPD), the more-dense cell subpopulations were more stress resistant, formed a more durable cell wall, and accumulated more glycogen and trehalose than the less-dense subpopulations. Indeed, the accumulation of glycogen and trehalose is necessary for the formation of a more-dense cell subpopulation^18^, although it is not the main determinant of cell density^19^. Trehalose is not only a reserve carbohydrate, but also an important low molecular weight stress-protective molecule that promotes resistance to various stresses^11^. Remarkably, glycogen and trehalose levels were significantly lower in both subpopulations of liqYPD compared to the corresponding subpopulations in colYPD, colGM and liqGM, whereas the resistance of these cells to Zymolyase treatment and heat stress was comparable to the other culture conditions. Overall, all 7 stationary-phase cell subpopulations were more stress-resistant and accumulated more glycogen and trehalose compared to exponentially growing and postdiauxic cultures, thus exhibiting characteristics typical of quiescent cells in stationary phase; only the intensity of these characteristics differed between more- and less-dense cells. This is consistent with other results showing that cells in stationary phase cultured under different conditions of nutrient starvation exhibit quiescence-specific features to varying degrees^15,20^. For example, a similar or even greater difference in storage carbohydrates, as observed here between cell subpopulations, was observed between stationary-phase cultures deprived of nitrogen, sulphur or phosphorus^20^.

### Common differences between more-dense and less-dense cells include some metabolic characteristics

Some metabolic traits (e.g., pyruvate decarboxylase and glycerol kinase activities and expression of some metabolism-related genes *ACS1, POX1, MET17*, and *ATO3*) were consistently higher in more-dense cell subpopulations regardless of carbon source and lifestyle (i.e. growth in colony vs. liquid culture). However, their possible connection is unclear. Pyruvate decarboxylase activity is tightly controlled by glucose availability^21^. This enzyme directs the glycolytic product, pyruvate, to form the overflow metabolites acetic acid and ethanol, both of which can be exported to the extracellular environment and act as age-promoting metabolites^22–24^. Glycerol kinase, on the other hand, is repressed by glucose and allows the utilization of another overflow metabolite, glycerol. Glycerol promotes longevity, as its excretion to the medium increases the chronological lifespan^24,25^. Differences in production and consumption rates, and possibly in the sensing of these ageing-related exometabolites, may be one of the bases for diversification between more- and less-dense cell subpopulations^26^.

### Different types of more-dense and less-dense stationary-phase cells show specific differences in their metabolic properties

Several cell properties were condition-specific, such as respiratory capacity and certain TCA cycle-related enzyme activities (citrate synthase, isocitrate dehydrogenase, and glutamate dehydrogenase), which were higher in more-dense cells than in less-dense cells in both glucose-grown populations (colonies and liquid culture), but lower in more-dense cells than in less-dense cells in GM-grown colonies. Interestingly, respiratory capacity was comparable in all more-dense cells and its striking difference concerned less-dense subpopulations: Respiratory capacity was low in less-dense YPD-grown cells (comparable to exponential liquid cultures in fermentative medium) and high in less-dense GM-grown cells (comparable to exponential liquid cultures in respiratory media). Thus, respiratory metabolism in subpopulations can be considered carbon source specific. This is consistent with studies showing that medium composition strongly influences the transcriptome, proteome and metabolome of stationary phase cells growing on these media^15,16^ and, furthermore, that different genes are required for long-term survival on different media^27,28^. On the other hand, some other metabolic traits (enzymatic activities of malate dehydrogenase, fumarase, and malate synthase) appear to be lifestyle-specific, as their differences between less- and more-dense cells depend on whether the cells originate from colonies or liquid cultures, regardless of the carbon source.

### Only one cell type is formed in liqGM

In contrast to the other populations, the cells in liquid GM medium did not differentiate into the two morphologically and physiologically distinct subpopulations and formed only one denser subpopulation (separated as fraction 3). These cells were morphologically and metabolically similar to the other more-dense cells. However, notable differences were also observed, particularly with regard to some of the enzymatic activities and the expression of specific GFP-tagged proteins. This suggests that the metabolism of liqGM cells also differs from the metabolism of other cell types, which could be partly due to the carbon source and partly due to their living conditions.

The question arises why the cell culture in liqGM does not differentiate into two cell types like all other cultures tested, including liqYPD with a similar cell lifestyle. A notable difference between liqYPD and liqGM cultures is the diauxic transition from fermentation to respiration that occurs in liqYPD and not in liqGM. It is therefore possible that this marked change in metabolism from fermentation to respiration is related to diversification of cells into two subpopulations in liqYPD. The accumulation of glycogen and trehalose, a characteristic feature of the more-dense cell subpopulations, is closely related to the glucose concentration in the medium. The production and activities of enzymes involved in glycogen and trehalose biosynthesis are controlled by glucose-sensing pathways^29^ and glycogen and trehalose accumulation increases during the diauxic shift and under conditions of caloric restriction^20,30^. During the diauxic shift, glycogen accumulation begins when the glucose concentration drops to about 50% of the initial concentration (i.e. long before glucose is fully depleted)^20^. Previous studies have shown that the majority of cells in the more-dense liqYPD subpopulation are virgin cells^6^, i.e. cells that have arisen during the last cell division. It is therefore likely that these cells arose under conditions where the glucose concentration in liqYPD is already low^31^. Thus, these virgin cells have never lived under conditions of high glucose concentration, and although they are unlikely to exhibit the characteristics of a more-dense subpopulation immediately after birth, their metabolism and regulations may predispose them to develop into a more-dense cell type. In contrast, a large proportion of other replicatively older cells present in the liqYPD population^6^ have undergone a diauxic switch from fermentation to respiratory metabolism, which could inhibit further development into more-dense cells by an as yet unknown mechanism. In contrast, cells cultured in a non-fermentable GM do not come into contact with glucose from the beginning of cultivation and do not undergo a switch in metabolism from fermentation to respiration, so their development into a more-dense cell type would not be inhibited according to this hypothesis.

In contrast to liqGM, diversification into two subpopulations occurs in colonies growing on GMA, although, similarly to liqGM, there is no diauxic switch from fermentation to respiration metabolism. In contrast to unstructured liquid cultures, there are different gradients of nutrients, waste products, exometabolites, and/or signalling molecules in colonies, and there are interactions between subpopulations, including the transfer of waste products and nutrients^17,32–35^. All of these create a structured environment that may be involved in cell differentiation. Therefore, the formation of individual cell types in colonies may be controlled in a different manner than in unstructured liquid cultures. Accordingly, in a number of cases, some regulatory pathways (even their combinations) function differently in differentiated colony subpopulations than in liquid culture cells^1,36^.

## Methods

### Yeast strains and media

*Saccharomyces cerevisiae* strain BY4742 (EUROSCARF) was used as the wild-type strain. C-terminal GFP fusion strains (Table 1) were derived from BY4742 by integrating yeGFP-KanMX transformation cassette from plasmid pKT127 into specific genomic locus^1,37^. Primers used for strain construction are listed in Supplementary Table S1. Cultures were grown at 28 °C in YPD (2% peptone, 1% yeast extract, 2% d-glucose) or GM (1% yeast extract, 3% glycerol, 1% ethanol) media, supplemented with 2% agar in the case of solid media.

**Table 1:** Strains used in the study.

Strain	Genotype	Source
BY4742	*MATα, his3Δ1, leu2Δ0, lys2Δ0, ura3Δ0*	EUROSCARF
Ato1p-GFP	*MATα, his3Δ1, leu2Δ0, lys2Δ0, ura3Δ0, ATO1-GFP-KanMX*	^45^
Ino1p-GFP	*MATα, his3Δ1, leu2Δ0, lys2Δ0, ura3Δ0, INO1-GFP-KanMX*	^1^
Pox1p-GFP	*MATα, his3Δ1, leu2Δ0, lys2Δ0, ura3Δ0, POX1-GFP-KanMX*	^1^
Met17p-GFP	*MATα, his3Δ1, leu2Δ0, lys2Δ0, ura3Δ0, MET17-GFP-KanMX*	^1^
Cit1p-GFP	*MATα, his3Δ1, leu2Δ0, lys2Δ0, ura3Δ0, CIT1-GFP-KanMX*	This study
Fbp1p-GFP	*MATα, his3Δ1, leu2Δ0, lys2Δ0, ura3Δ0, FBP1-GFP-KanMX*	This study
Acs1p-GFP	*MATα, his3Δ1, leu2Δ0, lys2Δ0, ura3Δ0, ACS1-GFP-KanMX*	This study
Nce102p-GFP	*MATα, his3Δ1, leu2Δ0, lys2Δ0, ura3Δ0, NCE102-GFP-KanMX*	This study

### Subpopulation fractionation

Yeast subpopulations were separated by density gradient centrifugation or Percoll gradient centrifugation as described^1,6^ from 7-day-old liquid cultures or 14-day-old colonies grown on YPD or GM. In density gradient centrifugation, yeast populations were fractionated by centrifugation (5 min, 140 g, 90° swing-out rotor, slow acceleration, no deceleration) in 45 ml of linear 15–35% sorbitol gradient. Fractions of 6 ml were collected from the top of the gradient, centrifuged (3 min, 1000 g), and the pellet was washed with 15 ml H_2_O, weighed and resuspended in an appropriate volume of water or buffer to obtain the required concentration. Fraction 1 (from top) containing the less-dense cell subpopulation, and fraction 3, containing the more-dense cell subpopulation, were used for subsequent analyses. Fraction 2 was discarded as it contained a mixture of both subpopulations. For Percoll centrifugation, we used Percoll density gradients prepared in 15-ml ultracentrifugation tubes according to the manufacturer’s protocol using Percoll diluted 10× with 0.15 M sodium chloride. Cell suspensions to be separated were placed on the gradient and centrifuged at 400*g* for 60 min.

### Respiratory capacity

For measurement of oxygen consumption, the isolated cells were resuspended in distilled water to a concentration of 100 mg/ml. 25 µl of the cell suspension was added to 1 ml of water in Mitocell MT200 measuring chamber connected to an oxygen meter (Strathkelvin Instruments) at 30 °C. After stabilisation of measured values, 55 µl of 96% ethanol was added using a syringe. The oxygen consumption rate was determined after stabilisation of oxygen concentration decrease from linear part of oxygen concentration curve (ca. 90–150 s after ethanol addition; Supplementary Fig. S1). Blank value of signal decrease in sample without cells was subtracted from measured values. No differences in onset of stable linear oxygen consumption rate (i.e. no lag) was observed between different cell subpopulations (Supplementary Fig. S1).

### Enzymatic activity assays

Enzymatic activities were measured in isolated subpopulations as described^9^. Briefly, 300 µl of cell suspension in extraction buffer (20 mM HEPES, pH 7.1; 1 mM dithiothreitol; 100 mM potassium chloride; 40 µl/ml protease inhibitor cocktail (Roche); and 1 mM 4-(2-aminoethyl) benzenesulfonyl fluoride hydrochloride (AEBSF)) was added to 200 µl of glass beads and disrupted 4 × 20 s at maximum speed in FastPrep (Thermo Fisher Scientific) with 1 min intervals on ice. The cell lysate was then clarified by centrifugation at 10 000 g for 10 min and diluted to the concentration of 2 µg protein per µl. Protein concentration was determined by Protein assay kit (Bio-Rad) with BSA as the standard. Enzymatic activities were determined in these crude extracts by following the kinetics of absorbance changes for 5 min in a spectrophotometer. Assay mixtures were composed according to the following published methods: citrate synthase^38^, NAD-dependent isocitrate dehydrogenase^39^, NAD-dependent glutamate dehydrogenase^40^, fumarase^41^, malate dehydrogenase^42^ and malate synthase^43^.

### Glycogen and trehalose determination

Glycogen and trehalose content were determined by a modified method developed by Masuko et al.^44^. 5 mg of isolated cells in 50 µl H_2_O was mixed with 200 µl 313 mM sodium carbonate and incubated at 90 °C for 4 h. Then 600 µl 0.2 M sodium acetate and 150 µl of 1 M acetic acid were added, and the sample was vortexed and centrifuged (2 min, 5000*g*). 200 µl of the supernatant was mixed with either 5 µl 2 U/ml trehalose (for trehalose assay; Megazyme) or 5 µl 50 U/ml amyloglucosidase (for glycogen assay; Sigma) and incubated overnight at 37 °C or 52 °C, respectively. Glucose released by enzymatic degradation was quantified using the glucose oxidase (GO) assay kit (Sigma).

### Stress sensitivity assays

For heat stress resistance testing, aseptically isolated cells were resuspended in water to a final concentration of 10 mg/ml. This suspension was then used as dilution 1 to make a tenfold dilution series from dilution 1 to dilution 10^−4^ in a microtiter plate. In each dilution step, 10 µl of the cell suspension was added to 90 µl of water. The plate was then placed in a PCR cycler and heated to 52 °C with slope of 0.2 °C/min and then incubated in 52 °C for 30 min. 5 µl of each dilution were spotted onto the YPD plate before incubation (time 0) and after 30-min incubation. The plates were photographed after 48 h and scored. The same suspension was used for the Zymolyase assay. Cell suspension (100 µl) was added to 800 µl of Zymolyase solution consisting of 20 mM potassium phosphate buffer, pH 7.8, 2 mM dithiothreitol and 5 U/ml Zymolyase 20 T (MP Biomedicals). After 1 min of incubation, the OD_600_ decrease was followed for 5 min and the slope of the OD_600_ decrease was calculated. The negative value of this slope is presented as a measure of cell wall susceptibility to Zymolyase.

### Fluorescence intensity measurement

The level of GFP-tagged marker proteins was measured as the intensity of GFP fluorescence using the FluoroMax spectrofluorimeter (Horiba Jobin Yvon). Fluorescence was measured in a cuvette containing 4 ml suspension of isolated cells with density adjusted to OD_600_ = 0.5. The excitation wavelength was set at 480 nm and emission was measured at 510 nm. The values obtained from a wild-type strain grown and manipulated under the same conditions were subtracted from the values measured in fluorescent strains. Fluorescence intensities of GFP-tagged marker proteins are shown in arbitrary units (1 unit corresponds to 1000 units of photodetector signal output).

### Statistical analysis

Presented values are means of at least three biological replicates. The number of replicates (n) is specified in figure legends. Mean values are presented with standard deviation. Statistical significance p-value was calculated using two-sample two-tailed t-test.

### Supplementary Information

Supplementary Information.

## Data Availability

All data generated or analysed during this study are included in this published article.
