# Supplementary material for: The characteristics of differentiated yeast subpopulations depend on their lifestyle and available nutrients
Source: Sci Rep. 2024 Feb 14;14:3681. doi: 10.1038/s41598-024-54300-9 (PMC10866891; doi:10.1038/s41598-024-54300-9)
Supplement: Supplementary file 1 — Supplementary Information. [file 41598_2024_54300_MOESM1_ESM.pdf]

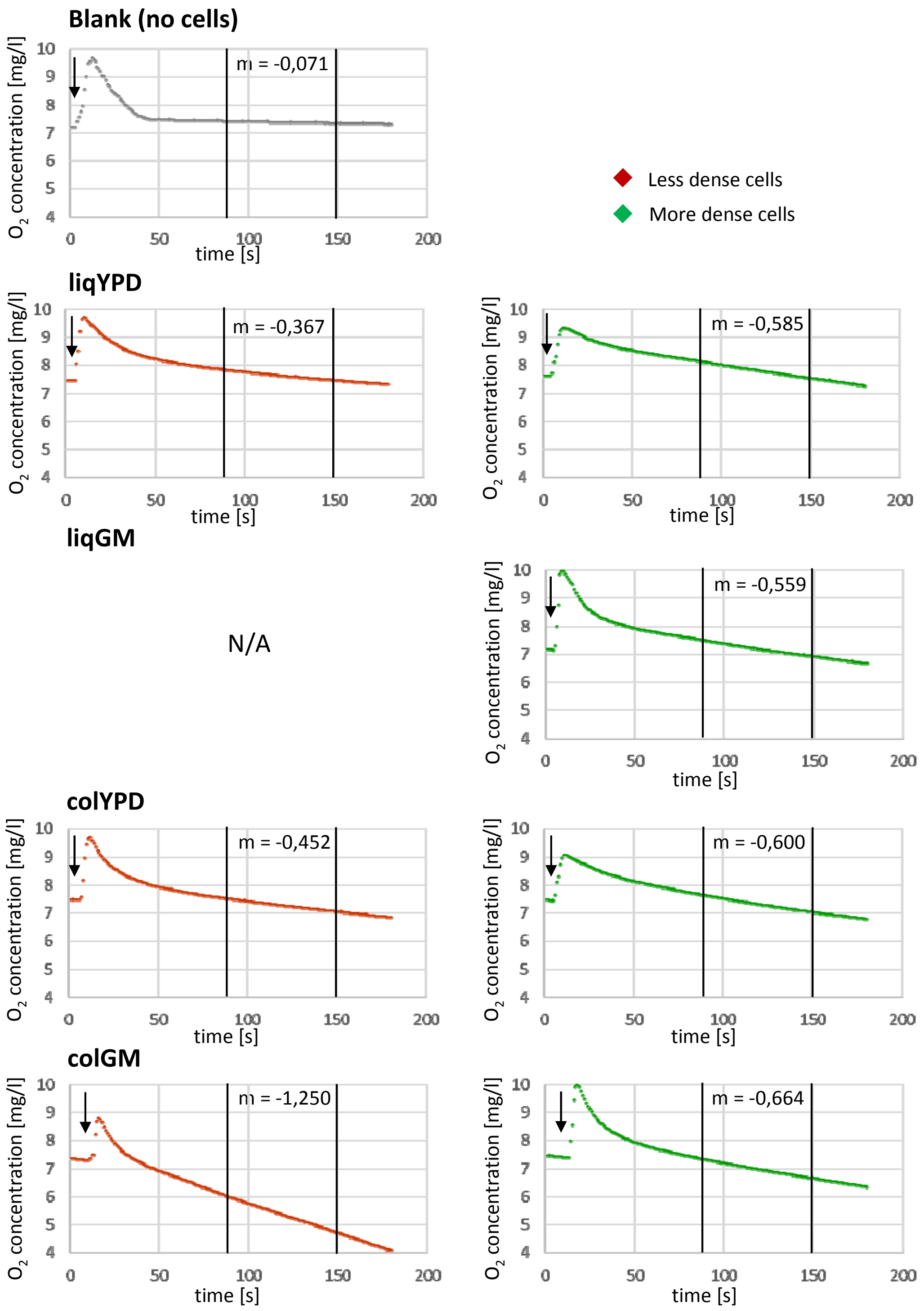

Supplementary Figure S1: Oxygen concentration curves. The addition of 55 μl of 96% ethanol is indicated by an arrow. A transient increase in oxygen concentration was observed after the addition of ethanol. The oxygen consumption rate was determined after the oxygen concentration stabilised and the decrease in oxygen concentration became linear. The slope (m) of oxygen consumption [mg O_2/l/min] by biomass was calculated from a 60 s interval approximately 90-150 s after oxygen addition (the interval is indicated by black vertical lines, the calculated slope values are given for each graph). The data show a representative sample for each cell type (red curve for less dense cells, green curve for more dense cells in each culture tested; the liqGM culture does not produce less dense cell type).

Table S1: Primers for the PCR amplification of cassettes from the plasmid pKT127 for the construction of genome-tagged yeast strains.

Strain	Forward primer	Reverse primer
Cit1p-GFP	ACCGAAAAATACAAGGAGTTGGTAAAGA AAATCGAAAGTAAGAACGGTGACGGTGCT GGTTTA	GTTTGAATAGTCGCATACCCTGAATCAAAAATCAA ATTTTCCTTATCGATGAATTCGAGCTCG
Fbp1p-GFP	GTTCTTCAGGTGAAATTGACAAATTTTTAG ACCATATTGGCAAGTCACAG GGTGACGGTGCTGGTTTA	ACTAAAGTACAGAACAAAGAAAATAAGAAAAGAA GGCGATCATTGAACTTATCGATGAATTCGAGCTCG
Acs1p-GFP	TCAAACCCTGGCATTGTTAGACATCTAATT GATTCGGTCAAGTTGGGTGACGGTGCTGG TTTA	AAAAAAAAAAAGTCGTCAATATAAAAAGGAAAGA AATCATCATTATCGATGAATTCGAGCTCG
Nce102p-GFP	CCAAGAGAAGAAGAACTGGCCAAGTCGG TGTTCCAACCATTTCCCAAGTCGGTGACGG TGCTGGTTTA	ATATTCTTAATTGAAAGTGGCGTAAAATTTAAGTT GGTGCGCTTCAATTTATCGATGAATTCGAGCTCG
